# Alcohol consumption and household expenditure on alcohol in a rural district in Vietnam

**DOI:** 10.3402/gha.v6i0.18937

**Published:** 2013-01-25

**Authors:** Kim Bao Giang, Hoang Van Minh, Peter Allebeck

**Affiliations:** 1 Institute for Preventive Medicine and Public Health, Hanoi Medical University, Hanoi, Vietnam; 2 Social Medicine, Public Health Sciences, Karolinska Institute, Solna, Sweden

**Keywords:** alcohol consumption, alcohol problems, alcohol expenditure, Vietnam

## Abstract

**Introduction:**

Alcohol use and alcohol-related problems are on the rise in low- and middle-income countries. Expenditure on alcohol is an important problem for families and communities and needs to be assessed.

**Aim:**

This study examines level of alcohol consumption and expenditure on alcohol in a district in Vietnam.

**Methods:**

A cross-sectional survey was conducted in a rural district in northern Vietnam. Multi-stage sampling was employed to randomly select participants from 20 communities and a town in the same district. One thousand five hundred and sixty-four adults (765 males and 799 females) aged 18–60 years were interviewed. Information about alcohol use as well as expenditure on alcohol consumption four weeks prior to the interview was gathered. Non-parametric tests and log-linear regression were employed to compare expenditure on alcohol consumption across socioeconomic groups.

**Results:**

The prevalence of alcohol use one month prior to interview was 35% (66% among men and 5% among women). The median alcohol consumption among those who reported use of alcohol in the week prior to the interview was 7.9 standard drinks. Excessive drinking (more than 14 standard drinks per week for men and more than seven standard drinks per week for women) occurred among 35% of those who used alcohol. Median expenditure for alcohol consumption during one month by those who drank alcohol was USD 3.5, accounting for 4.6% of household food expenditure, 2.7% of total household expenditure, and 1.8% of household income. The differences in alcohol consumption and expenditure between sexes and between socioeconomic groups are also presented.

**Conclusion:**

Our study confirms that alcohol consumption and alcohol-related problems are common among men in Vietnam. The share of alcohol expenditure in total household expenditure is substantial, especially among poor households. This should be considered an important public health issue, which needs to be taken into account in the alcohol policy debate.

Alcohol has a causal relationship with more than 60 medical conditions, and overall it causes 4% of total global burden of diseases (1, 2). Social and economic burden of alcohol consumption has also been well documented (2–5). The cost of alcohol problem has been estimated to be 1% of the gross domestic product in Canada and Australia (6), and 4% in New Zealand (7). In the United States, more than 20 cost components were included in a model to estimate economic cost of alcohol abuse, which consisted of health care cost, lost earning due to premature deaths, alcohol-related illness, crime and victim, and so on. The economic cost of alcohol abuse in the United States has increased by 3.8% every year (8). The social cost of alcohol consists of different types of alcohol-related costs, such as health service resources used, social work services, criminal justice, emergency services, economic cost, and human cost. The social cost of alcohol in Europe is estimated to be from 1 to 3% (9). Meanwhile, the alcohol-related tax revenue covers only a small part of the economic cost of alcohol abuse (4). Alcohol consumption also has a significant share in total household or consumer expenditure in many countries. It amounts to 5.8% of all consumer expenditure and 5.2% of household consumption expenditure in the United Kingdom (9).

**Policy recommendations**

In this study, we found that alcohol use and alcohol problems are very common among men in Vietnam. The share of alcohol expenditure in household expenditure is substantial, especially among poor households. Following are key policy recommendations:

- Alcohol use among men should be considered an important public health issue in Vietnam. Health communication and prevention programs should be conducted to raise community awareness and to minimize negative impacts of alcohol use in Vietnam.
- Development of alcohol policies and strategies should be given priority. Measures to reduce availability of, and accessibility to alcohol are necessary to decrease alcohol abuse, especially among men in Vietnam.

The level of alcohol consumption is reported to have decreased in developed countries, but it has been increasing in developing countries (2). In Vietnam, alcohol is widely available in the market and easily accessible (10). Alcohol drinking among men is traditionally and socially encouraged. Vietnam is a country with a high prevalence of alcohol use and alcohol-related problems (11–13). Among men, the prevalence of alcohol use during the 1-week period has been estimated to be 46% (12). A study that used the Alcohol Use Disorder Identification Test (AUDIT) – an instrument developed by the World Health Organization (WHO) to detect alcohol problems in Vietnam found the prevalence of alcohol-related problems to be 25.5% (13).

As the results of the increase of production in connection with economic growth in Vietnam, alcohol consumption has increased during the past decades (2). Thus, more economic and social costs would be attributed to alcohol consumption. In 2006, drunk driving caused 6.8% of traffic accidents (14). Alcohol use and alcohol-related problems are thus increasing public health concerns in Vietnam, as well as in many other low- and middle-income countries. For an informed debate on alcohol policy, it is important to take into account not only the well-known harmful effects on health but also other effects. Excessive expenditure on alcohol can be detrimental to families and communities. This study examines alcohol consumption and expenditure on alcohol consumption in a district in Vietnam.

## Methods

### Setting

This study was conducted in Thanh Oai district, Ha Noi, Vietnam, a typical rural district in northern Vietnam. The district had 20 communities, a town with a total population of 204,729 people, and a land area of 129.6 km^2^. The main economic activities were agriculture (47%), small scale industries and construction work (25%), and trade (27%). Around 3.7% of the households in this district were classified as poor according to official statistics.

### Design

A cross-sectional study was conducted in all communities and in the town of the district.

### Sample size and sampling

Sample size was estimated using the WHO formula for sample size to estimate prevalence of alcohol problems (*p =*0.25 is the prevalence of alcohol problems in previous studies (13, 15); relative precision = 0.1; significance level = 0.05). The required sample size was 1,153. This sample size is also adequate to compare expenditure on alcohol consumption in different socioeconomic groups. A multi-stage sampling was applied to select study subjects in this district. First, all 20 communities and a town in the district were selected. Second, Proportional Population to Size sampling technique was applied to estimate number of households to be interviewed from each community. To prevent non-response cases, in each community, we increased the sample size by 10–20 households based on its population size (3, 8% of total required sample size). The actual sample size was 1,600 households. Systematic sampling was used to randomly select households from each community. Finally, in each selected household, a person in the age range of 16–60 was randomly selected to be interviewed using Kish table. This is also called the Kish grid, a method using a pre-assigned table of random numbers to find the persons to be interviewed in a household (16). Eleven persons who were too sick or incapable of participating were excluded. Twenty-five persons could not be reached because of absence from home during the survey. Interviews were completed with 1,564 persons.

### Key variables

#### Alcohol consumption

Consumption of any kinds of alcoholic beverage. Beer at 4%, wine at 12%, 30% and 40% of pure alcohol are among the most common used beverage in this rural Vietnam. Information on alcohol consumption during 1 week and frequency of alcohol consumption during 4 weeks and 12 months prior to the interview was gathered.

#### Alcohol user

Those persons who consumed any kind of alcoholic beverage during 12 months prior to the interview.

#### Standard drink

The WHO definition of a standard drink was used to assess the volume of alcohol consumed during the week prior to the interview. A standard drink refers to the amount of about 12.6 g pure alcohol, which equals to 330 ml of 5% beer, 40 ml of 40% liquor, or 130 ml of 12% wine, and so on (17).

#### Alcohol consumption per person per year

Volume of alcohol consumption during a 1-week period, multiplied up to 1 year (4.333 weeks per month and 12 months per year).

#### Binge drinking

Consumption of more than five standard drinks in one sitting.

#### Excessive drinking

Intake of more than 14 standard drinks a week for men (more than two standard drinks a day) and more than seven standard drinks a week for women (more than one standard drink a day) (18).

#### Alcohol problem

An AUDIT score of 8 or higher was recognized as a social and health problem caused by alcohol. The AUDIT was developed by the WHO, and it has been used in many settings. The AUDIT consists of 10 questions, which have a possible maximum score of 40. The questions 1–8 may score 0–4 points, and question 9 and 10 may score 0, 2, or 4 points. The optimal cutoff level was found to be 8, and this has been proved to be reliable and valid to diagnose alcohol problems in many countries (17), including Vietnam (13, 15).

#### Household income quintile

The total income of a household during 1 year prior to data collection. The total household income from different sources and economic activities of all household members was assessed getting information from the head of the household.

#### Household expenditure during 1 month

Expenditure on different beforehand categories, including expenditure on 1) food and drinks; 2) electricity, telephone, and fuels; 3) education; 4) health care; and 5) others.

#### Expenditure on alcohol consumption

The entire money that an alcohol user spent on alcohol during a month. We did not take into account alcohol consumption, wherein the respondents did not have to pay.

#### Share of total household expenditure on alcohol expenditure

Expenditure on alcohol reported during 1 month prior to the interview divided by total expenditure of the household during the said month.

#### Share of household monthly food expenditure on alcohol expenditures

Expenditure on alcohol divided by the total expenditure on food and drink in the household in 1 month period.

#### Proportion of alcohol expenditure in total household monthly income

Expenditure on alcohol reported during 1 month prior to the interview divided by the household monthly income.

### Data collection

A questionnaire was developed to collect the information as mentioned above, including background information of study subjects, drinking frequency, average drinking volume, and expenditure on alcohol. The Vietnamese version of the AUDIT was integrated in the questionnaire to help identify cases with alcohol problems. The questionnaire was tested and revised to make sure all questions were understandable by participants. Face-to-face interviews were performed by 20 health staff from communal health stations in Thanh Oai. Interviewers were carefully trained to understand the purpose of the study, how to ask questions, and how to fill in the interview forms. Four staff members from district health centers and four research staff from the Hanoi Medical University were recruited for field supervision. Daily field supervision was done to ensure the quality of data collection.

### Data management and data analysis

Data were entered using the Epidata software. Check file was used to ensure logical information and reduce errors during data entry. STATA 10 was used to perform all analyses. Chi-square test was used to compare the prevalence of alcohol use and alcohol problems across groups. Non-parametric tests and linear regression were employed to compare expenditure for alcohol consumption across socioeconomic groups. To perform the linear regression model, logarithmic transformation was performed on four variables, including alcohol consumption, alcohol expenditure, share of alcohol expenditure in total household expenditure, and proportion of alcohol expenditure in household monthly income. In the log-linear regression models, number of household members, age group, marital status, education, occupation, and income quintiles were included as independent variables.

### Ethical clearance

This study was approved by the Ethical Committee of the Hanoi Medical University. Oral informed consent was gathered for each interview during data collection. Study subjects were informed about the study objectives, confidentiality of their individual information, and their right to refuse or stop the interview at any stage of the study.

## Results

Table 1 presents sociodemographic characteristics of the study subjects. Among 1,564 participants, 48.9% were men and 51.1% were women. Half of them (50.7%) were in the age range of 25–44 years, 74.7% were married, 72.8% of them had secondary level of education or higher, 44.6% of them were farmers, and only 16.2% of them were working in public offices or were students. Men and women had similar distribution of age and marital status. The proportion of farmers among women was higher than that among men (48.3% vs. 40.7%, *p =*0.0013), while the proportion of men who had at least high school level of education was higher than that of women (32.3% vs. 20.9%, *p <*0.0001).

**Table 1:** Background information of study subjects

		Males (*N =*765)		Females (*N =*799)		Overall	
				
Variables		*n*	%	*n*	%	*N*	%
Age group	18–24	168	22.0	175	21.9	343	21.9
	25–44	386	50.5	401	50.2	787	50.3
	45–60	211	27.6	223	27.9	434	27.7
Marital status	Other	196	25.6	199	24.9	395	25.3
	Married	569	74.4	600	75.1	1,169	74.7
Education level	Primary and lower	181	23.6	245	30.7	426	27.3
	Secondary	337	44.1	387	48.4	724	46.3
	High school and upper*	247	32.3	167	20.9	314	26.5
Occupation	Farmer	311	40.7	386	48.3	697	44.6
	Government staff and student	126	16.4	128	16	254	16.2
	Worker	195	25.5	117	14.6	312	19.9
	Others	132	17.2	166	20.7	298	19.1
Income quintile	Poorest*	132	17.3	182	22.8	314	20.1
	Second poorest	153	20.0	165	20.7	318	20.3
	Middle	167	21.8	158	19.8	325	20.8
	Second richest	148	19.4	151	18.9	299	19.1
	Richest	165	21.6	143	17.9	308	19.7

* The difference between two sexes is statistically significant by Chi-square test.

Alcohol use and alcohol problem were much more common among men. The prevalence of alcohol use during the 12-month period was 49.6% (82.9% among men and 17.8% among women). The prevalence of alcohol use during 1 week prior to the interview was 34.7% (65.5% among men and 5.3% among women, *p <*0.0001). The prevalence of excessive drinking was 24.4% among all men and was 0.4% among all women (*p =*0.0001) in the sample. The prevalence of excessive drinking was 35% among all alcohol users (37.3% among male alcohol users and 7.1% among female alcohol users; *p <*0.0001). The prevalence of binge drinking was 7% (14% among men and only 0.3% among women, *p <*0.0001). Alcohol problem was only recorded among males (24.1%) (Table 2).

**Table 2:** Alcohol use and alcohol problem

		Males (*N =*765)		Females (*N =*799)		Overall	
				
Variables		*n*	%	*n*	%	*N*	%
Alcohol consumption	Last 12 months*	634	82.9	142	17.8	776	49.6
	Last week*	501	65.5	42	5.3	543	34.7
Drinking frequency	None	131	17.1	657	82.2	788	50.4
	Monthly or less	159	20.8	128	16.0	287	18.4
	2–4 times a month	171	22.4	9	1.1	180	11.5
	2–3 times a week	133	17.4	0	0.0	133	8.5
	4 or more times a week	171	22.4	5	0.6	176	11.3
Average drinking level per sitting	1–2 drinks	571	74.6	796	99.6	1,367	87.4
	3–4 drinks	144	18.8	3	0.4	147	9.4
	5–6 drinks	39	5.1	0	0.0	39	2.5
	≥7 drinks	11	1.5	0	0.0	11	0.7
Excessive drinking	Among drinkers#	187	37.3	3	7.1	190	35.0
	Among study sample	187	24.4	3	0.4	190	12.2
Binge drinking	Never	658	86.0	797	99.7	1,455	93.0
	Less than monthly, %*	76	9.9	2	0.3	78	5.0
	Monthly	27	3.5	0	0.0	27	1.7
	Weekly or daily	4	0.6	0	0.0	4	0.2
Alcohol problem	AUDIT score ≥8*	184	24.1	0	0.0	184	11.8

* The difference between two sexes is statistically significant by Chi-square test.

# The prevalence is only calculated for alcohol users during 12 months prior to the interviews (Male alcohol users: 501; female alcohol users: 44).

The median of alcohol consumption during 1-week period prior to the interview among alcohol users was 7.9 standard drinks (8.9 standard drinks for males and 1.4 standard drinks for females) (Table 3). Non-parametric test comparing medians found that men significantly consumed more alcohol than did women (Table 3). Calculated as volume of alcohol per year, the average was estimated to 4.73 liters per person per year (9.37 liters per male and 0.33 liters per female).

**Table 3:** Number of standard drinks consumed by alcohol users during 1-week period

	Alcohol users		
	
Indicators	Male	Female	Overall
Quartile at 25%	3.0	0.8	3.0
Quartile at 50% (median)	8.9	1.4	7.9
Quartile at 75%	21.3	2.0	20.7

The mean expenditure on alcohol during 1 month among alcohol users was USD 6.5. More than 50% of the alcohol users spent at least USD 3.5, and 25% of alcohol users spent at least USD 7.1 for alcohol within a month.

On average, the monthly alcohol expenditure of an alcohol user accounted for 4.6% of monthly household food and drink expenditure, 2.7% of total household monthly expenditure, and 1.8% of household monthly income. Of the alcohol users, 25% spent at least 10% of the household food expenditure, 6% of the total household expenditure, or 4% of total household income on alcohol a month. Descriptive analysis shows that alcohol users in the middle income group had spent least money on alcohol (Table 4).

**Table 4:** Alcohol expenditure (in USD), share of alcohol expenditure in household food expenditure (%), in household expenditure (%), in household income among alcohol users during 1 month period

Variables	Income quintile	Median	IQR25	IQR75
Alcohol expenditure per month (USD)	Poorest	2.8	0.9	4.7
	Second poorest	3.5	1.9	5.9
	Third richest	2.4	1.2	4.7
	Second richest	3.5	2.1	7.1
	Richest	4.7	1.9	7.5
	Overall	3.5	1.6	7.1
Share of household monthly food expenditure on alcohol expenditure (%)	Poorest	5.7	1.8	13.3
	Second poorest	5.7	2.7	8.6
	Middle	3.6	1.9	8.0
	Second richest	4.8	2.7	10.0
	Richest	4.4	2.0	12.0
	Overall	4.6	2.2	10.0
Share of monthly household expenditure on alcohol expenditure (%)	Poorest	2.8	1.2	8.3
	Second poorest	3.7	1.9	5.7
	Third richest	2.1	1.1	4.8
	Second richest	2.9	1.6	6.1
	Richest	2.6	1.3	6.0
	Overall	2.7	1.3	6.0
Share of monthly household income on alcohol expenditure (%)	Poorest	4.7	2.1	11.5
	Second poorest	3.1	1.8	5.2
	Third richest	1.7	0.8	3.5
	Second richest	1.6	0.9	3.0
	Richest	1.1	0.5	2.2
	Overall	1.8	0.9	4.0

SD, Standard deviation; IRQ, Interquartile.

In the log-linear regression models, female alcohol users consumed significantly less alcohol than male. The married persons consumed significantly less alcohol than did unmarried persons. Those who had alcohol problems consumed significantly more alcohol than others. Those who had high school or higher education spent significantly less on alcohol, and had a lower share of household monthly food expenditure, household monthly expenditure, and household income each month on alcohol expenditure compared to those who had primary educational level or less.

Alcohol users in the highest income group spent significantly more money on alcohol than did the lower income groups. However, there was a clear and significant tendency that the better off groups had a lower share of household income on alcohol expenditure. There was no significant difference between income quintiles with regard to the share of household food expenditure and total household expenditure on alcohol expenditure.

Persons with alcohol problem spent more on alcohol, had larger share of alcohol expenditure in household food expenditure, total household expenditure, and household income. Those who were in the age range of 45–60 years had a lower share of household food expenditure and total household expenditure spent on alcohol during a month. No difference between occupation groups was found (Table 5).

**Table 5:** Intercepts and slopes for each variable, resulting from linear regression models for logarithm of monthly expenditure on alcohol consumption (USD), share of monthly household food expenditure, and household expenditure and household income on alcohol expenditure (%)

				Share on alcohol expenditure by (*n =*422)‡		
				
Variable		Alcohol consumption (*n =*528)#	Alcohol expenditures (*n =*422)‡	Household monthly food expenditure	Monthly household expenditure	Monthly household income
Number of household members		−0.03	−0.02	−0.12*	−0.11*	−0.03
Gender	Male					
	Female	−1.15*	−0.13	−0.19	−0.12	−0.18
Age group	18–24					
	25–44	−0.20	−0.25	−0.36	−0.35	−0.26
	45–60	0.05	−0.32	−0.56*	−0.58*	−0.41
Marital status	Other					
	Married	−0.65*	−0.24	−0.28	−0.21	−0.18
Education level	Primary and lower					
	Secondary	0.11	−0.17	−0.26	−0.25	−0.17
	High school and upper	0.01	−0.32*	−0.55*	−0.49*	−0.36*
Occupation	Farmer					
	Government staff and student	−0.19	0.14	0.19	0.08	0.16
	Worker	0.17	0.22	0.17	0.24	0.23
	Others	−0.04	0.08	0.04	0.08	0.08
Income quintile	Poorest					
	Second poorest	0.07	0.20	0.02	0.05	−0.47*
	Middle	−0.16	0.01	−0.24	−0.30	−1.03*
	Second richest	0.23	0.23	−0.05	−0.04	−1.09*
	Richest	0.36	0.40*	−0.04	−0.12	−1.54*
Alcohol problem	No					
	Yes	0.99*	0.71*	0.69*	0.68*	0.71*
Intercept		1.893	10.89	2.46	1.91	1.82
R^2^		0.218	0.15	0.13	0.14	0.28

# Only estimated among those who consumed alcohol during 1-week period.

‡ Only estimated among those drinkers who had to pay for alcohol during 1-week period.

* Statistical significant (*p <*0.05).

## Discussion

This study was conducted in a rural district in northern Vietnam. The population structure, educational status, economic activities, and economic and cultural status of this district are the same as several other districts in Vietnam. The findings are likely to reflect the situation regarding alcohol consumption and expenditure in rural Vietnam, although there are local variations.

We found that the 1-week prevalence of alcohol consumption was 34.7%, and the 12-month prevalence was 49.6%. The median of alcohol consumption among alcohol users was 7.9 standard drinks per week, and alcohol consumption estimated for a person per year was 4.73 liters of pure alcohol. This is slightly higher than what was recently reported by the WHO (3.8 liters per capita per year for persons aged 15 year old and above in Vietnam). Although Vietnam is not yet among the list of countries with high alcohol consumption levels, it is important to note that the level of alcohol consumption in Vietnam has increased at a rapid pace, by about 50% in the 5-year period between 2000 and 2005 (19). Although results from different studies in different time periods may not be comparable, together with other reports it seems clear that the prevalence of alcohol use has increased. For instance, in 2002, the National Health Survey found the 1-week prevalence of alcohol use to be 46% among men and 2% among women (12). In 2005, a study conducted in seven provinces reported 1-week prevalence of alcohol use to be 64% among men and 1% among women (11). Another study in rural Vietnam reported 12-month prevalence of alcohol use among male adults to be 87.3% and 10.2% among female adults (13). Alcohol drinking is reported to be on the rise in developing countries (2), including Vietnam (19).

The prevalence of alcohol problem was 24.1% among men, while none was found among women. This is supported by other studies reporting a similar distribution of alcohol problem among men and women (11, 13). The prevalence of excessive drinking in our study was 35% (37.3% among men and 7.1% among women). The prevalence of excessive drinking among men in our study is in line with the report of a study in seven provinces in Vietnam that 36% of men consumed more than three standard drinks per day. (11). Our study again indicates clear gender differences with regard to alcohol use, alcohol consumption, and alcohol problem (11–13). The prevalence of alcohol use among women in our study is slightly higher than reported in earlier studies from Vietnam (11–13). This suggests that alcohol use among women could have increased in Vietnam during the past few years. This can be seen as the result of socioeconomic development that contributes to close the gap between men and women, as has been observed in several other countries (20, 21).

Although we only took into accoun alcohol expenditure by a drinker from each household, we found that alcohol expenditure accounted for 4.6% of household food expenditure and 2.7% of total household expenditure. The share of alcohol in the household expenditure in our setting is slightly lower than what was reported in a country with higher consumption, such as the United Kingdom (19). In the United Kingdom, alcohol expenditure accounted for 5.2% in 2006 (9) and 3.4% in 2010 (3) of household expenditure.

In our setting, median of monthly expenditure on alcohol consumption of each individual alcohol user was USD 3.5. This is already about 30% of per capita income per month of a poor household in Vietnam, where the poverty line set by the government for the period 2006–2010 was VND 200,000 per capita per month (equal to USD 11.8) (22). Using the results of our study to crudely estimate total household expenditure on alcohol consumption in Vietnam, total expenditure on alcohol consumption among adults aged 18–60 years would be around USD 1.4 billion each year (about 60% of the 86 million population is in the age range of 18–60 (23), and the prevalence of alcohol consumption in that age group is 34.7%). These are crude estimations and may underestimate the consumption and expenditure in urban areas. Our figures nevertheless indicate an important burden on the economy of the household and that of the community.

It may seem obvious that those who had alcohol problems spent significantly more on alcohol and had larger share of alcohol in household expenditure and household income. We found that the highest income group spent significantly more on alcohol, but alcohol expenditure among the lowest income group accounted for a larger share of household income, thus increasing their already vulnerable situation. Also, Yen et al. (24) in a study from China found income, education, and other household characteristics to be significant determinants of household expenditure on alcohol. For a poor household, a large amount of income used for alcohol means less money available for food, health, and education. Thus, alcohol consumption could be one of the factors that put poor households at high risk of falling into a poverty trap.

Although Vietnam has already some policies, such as excise tax on alcohol products, mandatory business license to do alcohol trade, ban on alcohol sales in specific places, regulation on alcohol advertisement, ceiling on level of alcohol concentration in blood and breath of drivers, aimed at decreasing both alcohol supply and demand, these are far from being well implemented. Findings from this and other studies point to the need of improving and better enforcing current alcohol policy (11).

### Limitation

This study was conducted in rural Vietnam, so generalization of the results to other regions should be made with caution. In the urban settings, women are expected to have higher prevalence of alcohol use and consume more alcohol because in the urban areas women have more social activities and are more equal to men than women in the rura areas. Household income and expenditure could have been underestimated because these data were collected from the head of the households. Alcohol expenditure in the urban settings would be higher than in the rural settings because people seem to use more industrial alcohol products that are more expensive than home-made products often used in the rural settings (25), thus increasing the share of household expenditure and income on alcohol. Although in a household it is common for a person to buy alcohol to share with other household members, our study may underestimate alcohol expenditure because only money spent to buy alcohol by an alcohol user from each household was considered. Nevertheless, our results indicate alcohol expenditure accounted for a large part of household expenditure and income.

One may argue that alcohol abuse has been seen as a negative behavior, prompting the user to probably underreport the level of alcohol consumption. Recall bias may also be a problem when recall is requested. However, self-report methods are widely used, and they can be reliable and valid (26, 27). In our study, participants were requested to report volume of alcohol consumption only for a recall period of 1-week. Furthermore, we clearly explained the purpose of the study as well as the confidentiality of the private information. Interviews were conducted in a comfortable and private environment.

## Conclusion

Our study confirms that alcohol consumption and related problems are common among men in Vietnam. The share of alcohol expenditure in total household expenditure is substantial, especially among poor households, and it should be considered an important public health issue, which needs to be taken into account in the alcohol policy debate.

## References

[1] Room R, Babor T, Rehm J (2005). Alcohol and public health. Lancet.

[2] World Health Organisation (2004). Global status report on alcohol 2004.

[3] Collis J, Grayson A, Johal S (2010). Econometric analysis of alcohol consumption in the UK.

[4] Roeber J (2009). The human and economic cost of alcohol abuse in New Mexico, 2006. New Mexico Epidemiol.

[5] Minnesota Department of Health (2011). The human and cost of alcohol use in Minnesota.

[6] International Center for Alcohol Policies (1999). Estimating costs associated with alcohol abuse: towards a patterns approach.

[7] Cohen A, Kleiman K, Saraceno B (2002). World mental health casebook. Introduction.

[8] Harwood H (2000). Updating estimates of the economic costs of alcohol abuse in the United States: estimates, updates methods, and data: report prepared by The Lewin Group for the National Institute on Alcohol Abuse and Alcoholism.

[9] Institute of Alcohol Studies (2008). Economic cost and benefits.

[10] General Statistics Office, Ministry of Health, World Health Organisation, United Nations Children's Fund (2003). Survey assessment of Vietnamese youth.

[11] Health Strategy and Policy Institute (2006). Situation assessment of alcohol abuse in Vietnam (in Vietnamese: danh gia tinh hinh am dung ruou bia tai Viet Nam).

[12] Ministry of Health and General Statistic Office (2003). National health survey, 2001–02.

[13] Giang KB, Allebeck P, Spak F, Van Minh H, Dzung TV (2008). Alcohol use and alcohol consumption-related problems in rural Vietnam: an epidemiological survey using AUDIT. Subst Use Misuse.

[14] Tuan NN (2009). Alcohol related traffic accident and its prevention. Bureau for Road and Railway Traffic Security Ministry of Public Security.

[15] Giang KB, Spak F, Dzung TV, Allebeck P (2005). The use of audit to assess level of alcohol problems in rural Vietnam. Alcohol Alcohol.

[16] Kish L (1965). Survey sampling.

[17] Babor TF, John CH, Saunders J, Monteiro MG (2001). AUDIT – The Alcohol Use Disorders Identification Test – guidelines for use in primary care.

[18] US Department of Agriculture (2000). Nutrition and your health: dietary guidelines for Americans.

[19] WHO (2011). Global status report on alcohol and health.

[20] Simons-Morton BG, Farhat T, ter Bogt TFM, Hublet A, Kuntsche E, Gabhainn SN (2009). Gender specific trends in alcohol use: cross-cultural comparisons from 1998 to 2006 in 24 countries and regions. Int J Public Health.

[21] Wilsnack RW, Vogeltanz ND, Wilsnack SC, Harris TR (2002). Gender differences in alcohol consumption and adverse drinking consequences: cross-cultural patterns. Addiction.

[22] Prime Minister (2005). Decision No 170/2005/QD-TTg referring the issue of poverty line for the period 2006–2010. In: Vietnamese Government, editor.

[23] General Statistics Office (2006). Survey on population changes and family planning 1/4/2005: main findings.

[24] Yen ST, Jensen HH (1995). Determinants of household expenditures on alcohol.

[25] Giang KB (2012). Alcohol use in three provinces from three geographical regions in Viet Nam.

[26] Del Boca FK, Darkes J (2003). The validity of self-reports of alcohol consumption: state of the science and challenges for research. Addiction.

[27] Allen JP, Wilson VB, National Institute on Alcohol Abuse and Alcoholism (2003). Alcohol consumption measures. Assessing alcohol problems: a guide for clinicians and researchers.

